# Easier in Practice Than in Theory: Experiences of Coaches in Charge of Community-Based Soccer Training for Men with Prostate cancer—A Descriptive Qualitative Study

**DOI:** 10.1186/s40798-022-00424-z

**Published:** 2022-03-03

**Authors:** Kickan Roed, Eik Dybboe Bjerre, Julie Midtgaard

**Affiliations:** 1 The University Centre for Health Research, Copenhagen University Hospital, Rigshospitalet, Blegdamsvej 9, 2100 Copenhagen Ø, Denmark; 2 Mental Health Center, Glostrup, Copenhagen University Hospital - Mental Health Services CPH, Centre for Applied Research in Mental Health Care, Nordstjernevej 41, 2600 Copenhagen Ø, Denmark; 3 Department of Clinical Medicine, University of Copenhagen, Blegdamsvej 3B, 2200 Copenhagen N, Denmark

**Keywords:** Soccer, Exercise, Rehabilitation, Interview, Learning, Coaching, Non-professional, Community, Sport, Health promotion

## Abstract

**Background:**

Evidence suggests that community-based exercise programs and sports participation benefit long-term physical activity adherence and promote health in clinical populations. Recent research shows that community-based soccer can improve mental health and bone health and result in fewer hospital admissions in men with prostate cancer. However, little knowledge exists on what coaches experience, leading to a scarcity of knowledge on how to assist them in promoting and supporting the sustainability of programs. The purpose of this study was to explore the experiences of non-professional soccer coaches in providing community-based soccer training for men with prostate cancer.

**Results:**

We interviewed 13 out of 21 eligible non-professional soccer coaches in charge of delivering the Football Club Prostate Community program, which is community-based soccer training for men with prostate cancer at 12 local soccer clubs across Denmark. Qualitative content analysis, as described by Graneheim and Lundman, was applied to analyze the data using NVivo 12 software. We identified the five following overall categories with 10 subcategories on what the coaches experienced: (1) enabling training of a clinical population in a community setting, (2) dedication based on commitment, (3) coaching on the players’ terms, (4) navigating the illness, and (5) ensuring sustainability. Collectively, the findings suggest that, while the coaches felt adequately prepared to coach, their coaching role developed and was refined only through interaction with the players, indicating that coaching clinical populations may be easier in practice than in theory and a potentially transformative learning experience.

**Conclusions:**

Non-professional soccer coaches in charge of delivering soccer training for men with prostate cancer value being educated about specific illness-related issues. Initial concerns about how to coach a clinical population disappeared once the coaches engaged with the players and developed their own team norms and illness management strategies. They also gained a broader perspective on their own lives, which they valued and would not otherwise have achieved by coaching a healthy population. Our study indicates that sustainable implementation and the program’s sustainability can be promoted and supported through additional formal, easily accessible communication with trained health professionals and by networking with peer coaches.

**Supplementary Information:**

The online version contains supplementary material available at 10.1186/s40798-022-00424-z.

## Key Points

- Investigation of the experiences of non-health professionals can provide valuable insight into safe and sustainable (low-cost) implementation and dissemination of community-based exercise programs that are fundamental to long-term exercise adoption and adherence.
- Providing theoretical, formal education for coaches about illness-related issues is essential, but only in practice do they develop their own style and norms and value interacting with a clinical population, which offers a new perspective on life, providing coaching with new meaning.
- Sustainability of rehabilitative and/or health-promoting community-based exercise programs can be supported by more formal, easily accessible infrastructure and communication with hospitals and trained clinicians and through networking with peer coaches.

## Background

Accumulating evidence shows that increased physical activity, ranging from prevention to rehabilitation and palliation, for people with chronic disease, including various types of cancer during all stages of disease, provides substantial health benefits [1]. Physical activity is associated with a decrease in mortality from all causes, including cancer-specific mortality in people with prostate [2, 3], breast [4, 5], and colorectal cancer [6, 7]. Moreover, exercise training has also shown a positive effect on cancer-specific fatigue [8, 9] and cancer-specific quality of life [8, 10]. However, exercise-based rehabilitation is not standard care for people with cancer, which is why most people with cancer are not offered physical activities or exercise [11, 12]. Thus, a large number of people with cancer state that they have an unmet need for exercise-based rehabilitation [13]. When offered, supervised exercise-based rehabilitation programs can provide safe learning environments [14], yet supervised interventions are often gym-based, time-limited and rarely in line with user preferences [15]. Consequently, the potential to persist with exercise long-term following exercise-based rehabilitative interventions remains limited [16, 17]. Thus, there is a burgeoning appreciation of the need to promote and implement novel interventions that effectively and consistently increase and maintain levels of physical activity in people with cancer [15]. Notably, increased attention is given to exercise-based rehabilitative programs that are community-based in order to identify sustainable, cost-efficient ways of organizing physical activities for clinical populations that increase long-term engagement [18–20]. Sports participation in natural settings (e.g. recreational soccer) is viewed as a usable public health strategy that promote engagement in physical activity [21–24]. Moreover, epidemiological studies have shown that sports participation is associated with a reduction in all-cause mortality and protects mental health compared to non-participation [21, 25]. Especially participation in club sports and team-based sports has been shown to be effective in improving mental and social health [25].

We developed the Football Club Prostate Community (FCPC) initiative involving implementation and evaluation of recreational soccer training for men with prostate cancer in local soccer clubs across Denmark [26–28]. FCPC was developed based on existing infrastructure for physical activity offering easy-access, time-unlimited, and low-cost community-based exercise training [29]. The training is supervised by non-professional soccer coaches with no requirements for prior clinical experience or training as healthcare professionals [30]. However, all FCPC coaches completed a full one-day course on the general principles and delivery of recreational soccer for men with prostate cancer, including information on treatment regimens, side effects, and contraindications for physical activity for this group [18]. The curriculum, which also includes principles on how to support a sense of community among the participants [30], was implemented by the Danish Football Association. Evaluation of the effectiveness of FCPC suggested that the program is feasible and improves mental health [18]. The study showed that 59% of the men joined their local soccer club after completing the six-month trial period [18], indicating a long-term engagement in physical activity. Continued participation beyond six months results in improved hip bone mineral density and fewer hospital admissions [31].

While the results of FCPC hold promise for the dissemination of recreational sports in cancer rehabilitation, little research exists that can provide an understanding of what it means for non-professionals to carry the responsibility of delivering exercise training for a clinical population in the community.

Against this background, the *objective of this study* was to explore the experience of non-professional soccer coaches in providing community-based soccer training for men with prostate cancer. A *secondary objective* was to identify key recommendations of relevance for future planning, implementation, and dissemination of community-based training programs for clinical populations supervised by non-professionals.

## Methods

### Study Design

The current study applied a qualitative descriptive design involving researcher triangulation and use of semi-structured individual interviews. This study reported according to the Consolidated Criteria for Reporting Qualitative Research (COREQ) checklist [32] (Additional file 1: Table S1).

### Sampling

Because of the limited number of FCPC coaches in Denmark, a convenience sample was chosen to secure enough participants for the study. In November 2020, one of the researchers (KR) emailed an invitation containing information about the nature of the study to 24 potential participants, i.e., non-professional FCPC coaches in local soccer clubs across Denmark registered with the Danish Football Association. The only criterium for inclusion was current or previous experience as an FCPC coach who had completed the FCPC training course. As a result, three potential participants were excluded because they had been or were a player on a team rather than a coach. Of the remaining 21 potential participants, 13 wished to participate and eight did not respond. Data saturation defined the final sample size [33]. As information redundancy was identified already around the ninth or tenth interview, we chose not to contact the eight non-responders again. Moreover, the coaches who did not respond to the initial invitation would not have added to the variation of the sample; in other words, they did not differ from the responders for type of club, age, or years of coaching experience.

### Data Collection

Semi-structured individual interviews were carried out over the phone by KR (female investigator, full time research assistant), who has previous experience in conducting qualitative telephone interviews. KR did not know the informants previously and had not been involved in earlier studies related to the FCPC program. Individual interviews were chosen to enable the sharing of personal knowledge, acquired skills, and experiences in relation to delivery of the FCPC program, including the generation of personal reflections on one’s own role and responsibility. Further, telephone interviews were conducted due to the large geographical distance between the different coaches and the researcher but also because the assumption was that the interviews would not evoke strong feelings and/or stories.

To the best of our knowledge, no research is available on how non-professional coaches experience delivery of exercise-based rehabilitative programs for clinical populations in the community. As a result, an interview guide was developed based on questions and concerns previously raised by experts in e.g., human physiology, soccer, prostate cancer, and male psychology and by other stakeholders, e.g., urology nurses, non-professional soccer coaches, and men with prostate cancer, who informed the development of the educational program for FCPC coaches [30]. Their questions and concerns addressed the coaches’ qualifications, structure of the training, prevention of injuries, creation of non-patient environments, social inclusion as opposed to stigmatization, and positive competition [30]. The interview guide also covered issues addressed in current articles and debates in the scientific exercise-oncology literature on the implementation, dissemination, and sustainability of physical activities for people with cancer [11, 12, 15, 19, 20]. Finally, an expert in the organization and management of club soccer employed by the Danish Football Association, which implemented FCPC, critically revised the interview guide.

The interview guide consisted of open-ended questions as well as more focused questions on the coaches’ general experience as FCPC coaches, including their perspectives on their role as an FCPC coach, their motivation to become an FCPC coach, and their specific experience coaching a clinical population, as well as on the differences in relation to any previous experiences coaching non-clinical populations. The open-ended questions also covered their views on the need for additional skills, knowledge, or support, but also on injury prevention, support from their local soccer club, and barriers to FCPC, not to mention recommendations and advice for anyone considering coaching a clinical population. The interviewer asked probing questions such as: “Do you have other examples of that?” or “Could you elaborate on that?” to elicit any further information relevant to the study objective. The interviews ended with questions on demographic data, reflecting the variability of the sample (Additional file 2: Table S2). All thirteen interviews were conducted between November 30, 2020 and December 18, 2020, at a time convenient to the participants. Audiotaped, the interviews lasted 20 to 51 min, or an average of 32 min.

During the data collection process, as KR interviewed the participants and JM (female investigator, full time researcher) listened to the audiotapes, we focused on the emergence of new perspectives and exceptions to promote data saturation and to determine when it was reached. Once we agreed that redundancy had been achieved, we decided to finalize the recruitment of informants. Additionally, member checking was continuously performed during interviews to determine the credibility of the results [33, 43].

### Data Analysis

The interviews were transcribed verbatim in accordance with conventions described in a transcription manual [35]. Qualitative content analysis, as described by Graneheim and Lundman, was applied to analyze the interview data as an inductive approach is well-suited for studies with a limited amount of research available [36]. NVivo 12 (QRS International, Melbourne, Australia) was used to assist in data management.

KR and JM initially read and reread the interview transcripts separately before jointly discussing them to obtain a sense of the complete data material. Subsequently, KR identified and extracted meaning units consisting of sentences or paragraphs related to the study objectives. Next, KR condensed the meaning units, though preserving their meaning. The condensed meaning units were then compared and labeled with descriptive codes during detailed discussions with JM. Next, KR and JM compared, discussed, abstracted, and sorted the codes into 10 subcategories according to their distinct nature in content, differentiating them from other groups of codes. We further abstracted and interpreted the mutual interrelationship and differences between the content of the subcategories, which led to the creation of five overall categories. Codes, subcategories, and categories were continuously compared with the original transcripts. Table 1 provides examples of the condensation-abstraction process.

**Table 1:** Example of the condensation-abstraction process for the category: Coaching on the players’ terms

Meaning unit	Condensed meaning unit	Code	Subcategory	Category
*Example 1* Because they can easily get something out of it at their level, and our most important task as coaches is to identify that level and help them get something out of it [C11]	Important task to find a balance and ensure that everyone, despite differences in age and physical ability can participate in sports	Important aspects of the role as an FCPC coach	Adjusting the program to fit the players’ needs and to support adherence	Coaching on the players’ terms
*Example 2* And that socially we also have time to talk to each other, on the field that is. It’s not so much a question of being the winner that counts when we play against each other, it’s more the togetherness that matters [C3]	Social cohesion is incredibly important, which means that as a coach you also have to make the time for it during practice	The social community	Facilitating social community	

### Ethics

In accordance with Danish law, due to the qualitative nature of the present study, formal ethics committee approval was not required. In addition to receiving written information about the nature of the study prior to participation, all participants provided informed oral consent and were guaranteed anonymity before telephone interviews began. The study was conducted in accordance with the Declaration of Helsinki.

## Results

### Sample Characteristics

This study included 13 FCPC coaches (all male) representing 12 different soccer clubs and FCPC teams across Denmark. They served as FCPC coaches from one to eight years, or an average of 2.68 years each. Ten currently coached but three no longer did so. Two of the included coaches were FCPC soccer players who had been asked to coach when a former FCPC coach resigned. Table 2 presents participant characteristics.

**Table 2:** Participant characteristics (*n* = 13)

Characteristic	*n* (unless stated otherwise)
FCPC coach	
Current	10
Prior	3
Age, mean in years (min–max)	60 (26–85)
Time as FCPC coach, mean in years (min–max)	2.68 (1.0–8.0)
Prior FCPC soccer player	2
Prostate cancer diagnosis	3
Previously played soccer	10
Previously coached soccer	10
Inhabitants of the city of the local club	
> 200,000	6
< 60,000	7

### Characteristics of Clubs/Teams

Local soccer clubs have a high degree of autonomy regarding the organization and execution of FCPC, which is reflected in our sample. Four of the included coaches represented FCPC teams that comprised various groups of men, e.g., healthy seniors or men with other types of chronic illnesses, while one FCPC coach permitted two men to join who had approached him even though they had other types of cancer because no other community-based rehabilitation interventions were available.

### Analysis of Findings

The results of the analysis resulted in five overall categories and 10 subcategories (Table 3).

**Table 3:** Results of the analysis

Subcategories	Categories
Preparing to coach a clinical population Convenience of using well-established soccer clubs	Enabling training of a clinical population in a community setting
Motivating factors Personal investment and gain	Dedication based on commitment
Adjusting the program to fit the players’ needs Facilitating social community	Coaching on the players’ terms
Illness in the foreground vs. illness in the background Managing illness progression and deaths	Navigating the illness
Unappealing infrastructure Finances and retaining coaches	Ensuring sustainability

### Enabling Training of a Clinical Population in a Community Setting

While the FCPC coaches generally expressed feeling confident in their delivery of soccer training for men with prostate cancer, largely due to their extensive experience as soccer coaches, they believe that it does require some form of formal training to coach a clinical population in the community.

#### Preparing to coach a clinical population

Across interviews, the coaches described how they—before taking part in the one-day FCPC course and before meeting the men—were concerned about what it would be like to coach a clinical population:

C3 Can you joke about the disease? What can they actually do physically? (…) What if someone gets sick along the way? How the hell am I supposed to deal with that as a coach?

However, participants described feeling adequately prepared after completing the one-day FCPC course, which they agreed should be mandatory. Specifically, coaches described feeling reassured that they would be able to deliver a product that respected the population’s specific needs:

C2 They could otherwise just as well play on a regular team, on a regular pitch, as a soccer player in any other club.

A few coaches said that issues related to disease, side effects, and potential deaths made them apprehensive:

C5 The healthcare staff has to be on watch; they have to be sure about what we can do. And that might make you a bit nervous, causing you to think, ‘Oh no, what might I do wrong?’

Nevertheless, participants described how their worries disappeared once they met the players, who, according to participants, showed up in high spirits eager to play soccer, leading to an experience shared by many coaches, who stated that FCPC is easier in practice than in theory.

Some described staying in contact with other FCPC coaches or the doctors and nurses they met during the FCPC course and appreciated the possibility of asking questions in case of any uncertainty and/or doubts about prostate cancer.

#### Convenience of Using Well-Established Soccer Clubs

Participants described the fact that FCPC was designed and delivered in collaboration with and in the context of well-established local soccer clubs as essential to providing legitimacy and securing the necessary practical resources of value to the sustainability of the program and the coaches’ responsibilities, for instance, changing facilities and booking the football pitch:

C2 There’s a clubhouse run by volunteers who make sure that when we practice, even though no one else is there, there’s always someone available to serve coffee, tea, beer, or other beverages – EVERY single time!

The coaches also described that the embeddedness of the FCPC program within established local clubs constitutes a vital source for attracting coaches to FCPC because most of them had been affiliated with their club for decades prior to FCPC. Only two coaches said that their FCPC teams largely functioned as isolated silos, while the others talked about how their FCPC teams were an embedded, integral part of the local club, which the coaches believed positively influences the players’ perception of being taken seriously, as well as their own:

C3 I’m a busy coach around here, so it’s great to know that the people making the decisions also think that it has a purpose and think that it’s important that we’re part of the club’s makeup.

### Dedication Based on Commitment

Regarding their motivation to take on the role of coach, participants described a general feeling of being dedicated to improving people’s physical abilities. Through FCPC and a commitment to the men on their team, they experienced an unprecedented sense of duty and witnessed how the players benefited and found joy in the game, which, in turn, supported the coaches’ ongoing dedication.

#### Motivating Factors

Several coaches reported that they were headhunted to participate when the FCPC team was established at their local club. Presented with the idea of coaching men with prostate cancer, participants described an aspiration to provide people of all ages with opportunities to participate in and to experience the benefits and enjoyment that derive from physical activity:

C11 This is what sports is all about, that you can differentiate to find out what’s suitable for everything and everyone. So, I just hope that more people would like to do sports, also the elderly, and that they won’t feel intimidated by it, so they don’t just sit at home on the couch and get older than they have to be.

The retired coaches also said that they were motivated by wanting to have more to do during retirement while also taking care of their own need to exercise, while others found that being or knowing someone diagnosed with prostate cancer was motivating. Some of the younger coaches mentioned that writing FCPC coach on their resume might be of value later in their professional career.

#### Personal Investment and Gain

The participants said that they invested a considerable amount of time in and were committed to FCPC, including keeping in constant contact with the players, driving long distances to and from practice at the soccer club, socializing before and after practice, and participating in social events. The resources they invest are described as being repaid as good experiences, new friendships, and personal enrichment because the player’s excitement was contagious, offering a new perspective on daily life, insights, and an appreciation of the gifts life has to offer:

C10 You get a look at daily life when you work with both the elderly and a group of people who are ill, people who just show up for practice with enthusiasm and high spirits, despite their circumstances.

### Coaching on the Players’ Terms

The coaches, who described FCPC as having two equal purposes, i.e., to facilitate exercise and to facilitate community, said that the overall responsibility of the coach is to guarantee that the program is executed according on the players’ terms to promote adherence.

#### Adjusting the Program to Fit the Players’ Needs

Balancing the differences in level, age, physical ability, and mental state, while giving everyone the opportunity to improve their level of fitness, is described as an important and exciting coaching challenge. Physical exercise is highlighted as important due to both the health and social benefits:

C9 I’ll be damned if it doesn’t give some of them a sense of pride when they say that they are soccer players.

The coaches described initially adhering strictly to the FCPC training manual, though later using it more as a framework that they added features to from their catalogue as experienced coaches to meet the players’ preferences and thus ensure continued motivation:

C3 The players would rather mainly play soccer then do too much fitness training, so I adapt what I do in relation to that.

Continuous adherence to the manual took place chiefly to reduce the risk of injuries, which the coaches primarily stated to attribute to the men’s advanced age rather than their cancer diagnosis. Some coaches introduced standing in a circle before practice to discuss any aches and pains and the status of their illness. In this context, coaches described their role as less authoritarian than it would have been in case of players who were healthy and/or younger. Specifically, coaches said that they trusted that players knew what was best for themselves and that their foremost task was to meet the participants on their terms:

C13 You can just show up tired and say, ‘My treatment has knocked me flat, but I’d like to take part as best I can’.

#### Facilitating Social Community

According to the coaches, the social community is the most important aspect for the players, which is why they consider it their most prominent goal to adapt the training and prioritize activities that support the players’ sense of camaraderie. The coaches described how they consciously make an effort to support an inclusive environment so that the community can grow, for instance, by encouraging people to meet for breakfast before practice or to sit and relax together afterwards to talk over a cup of coffee or a beer.

The coaches believed that they bear a special responsibility for creating an atmosphere of mutual caring, both in terms of illness and soccer:

C6 One guy showed up who was just past 60, and he had never played soccer in his entire life (…) he showed up with NO experience whatsoever! And already after a few months, he practiced and received special instruction (…) so we also actually taught the guy to play football. And the support that the others gave him! Wow, he was showered with PRAISE. People cheered wildly whenever he didn’t fall and managed to kick the ball to another player.

### Navigating the Illness

The amount of prominence the disease is given and how the related challenges are handled varies between coaches, but also changed as they get to know the players and found their personal coaching style. A central cause for concern among the coaches was how to deal with a worsening of the disease and death.

#### Illness in the Foreground Versus Illness in the Background

The coaches distinguished between their job and that of the healthcare professionals. They respected the healthcare staff’s expertise and did not wish to intrude on it, preferring not to burden the players with illness-related talk in an effort to balance how much attention the illness was given. Some coaches described consciously holding back but mentioned talking about the illness was a natural corollary:

C4 For the most part, the guys manage on their own (…) when you hang out in the changing room, or you have a beer afterwards and chat, then lots of things come up.

Other coaches mentioned how they actively supported conversations about illness and that an exchange of experiences took place, for example, on walk and talks:

C5 Some people have to wear adult briefs, some have had a sphincter operation, and others have a stoma, those are the breaks when you practice with us, but you have to create room for people to also be able to show concern.

Coaches with teams solely comprising individuals with prostate cancer described a strong bond existing between the players, where nothing is off limits and where jokes are allowed about weight gain, impotence, and incontinence, which means that players would not hesitate to shout, “Damn this diaper is rubbing me raw!”. The same openness does not appear to be present or possible in clubs where the FCPC includes players who do not have prostate cancer:

C8 That’s one of the reasons why some of the guys already put on their workout clothes before they arrive.

#### Managing Illness Progression and Deaths

The coaches indicated that they were not generally concerned about the men’s illness, but that when a player was considerably affected by side effects or disease progression, they became somewhat uneasy and maintained extra contact with the player. All coaches voiced concern about possible deaths and how to handle them. Those who experienced deaths described dealing with them together with the players. Some show a sign of respect by sending flowers, attending the funeral and/or performing a ritual to pay tribute to and remember the player who died:

C13 They [the players] show up with a bottle of bitter and glasses, which is something they had planned if one of them died, in order to gather before practice to have a little drink in memory of the one who passed away.

### Ensuring Sustainability

Coaches pointed out limits to the sustainability of FCPC, which they largely view as being beyond their influence. This is a source of frustration, prompting them to call for improvements that would allow them to focus their energy and resources on the act of coaching.

#### Unappealing Infrastructure

All coaches emphasized continuous recruitment as a necessary requirement for the longevity of teams due to the fact that players continuously dropout or are absent due to treatment and side effects.

In FCPC clubs located far away from a prostate cancer treatment center or hospital, the coaches explained that recruiting enough men with prostate cancer was nearly impossible, leading them to invite other men to participate:

C1 There were usually about 4-6 players at practice, which is a difficult number if you want to keep practices fun (…) I believe it would have stopped again, causing people to lose the desire to play.

The coaches would like to see guidelines and specific opportunities for how to strengthen the referral and recruitment of new players. In the absence of more specific, formal support and/or instructions in this regard, the coaches described how they discovered and developed alternative ways to recruit, such as regional or national news programs or the local newspaper reporting on FCPC.

Across interviews, the coaches underlined that it is crucial to establish a connection to the local cancer rehabilitation coordinators and the nearest hospital’s urological and oncology wards, in addition to providing information for nurses and doctors about FCPC to ease the referral and recruitment of players. Moreover, coaches believed that the authority of healthcare professionals aids in legitimizing FCPC:

C11 Referrals from a clinician might remove some of the dangers of going to a club and playing soccer.

#### Finances and Retaining Coaches

The coaches voiced concerns about the sustainability of funding for the FCPC program. Specifically, they explained that FCPC was initially supported by temporary funding, which meant they were currently dependent on the willingness of their local soccer club to provide ongoing financing.

Some also touched upon the difficulties that exist in terms of recruitment and retention of coaches. Since many coaches are still on the labor market, it is problematic for them to coach because FCPC takes place during the daytime, which is when the target group (60–85 years of age) is most willing to participate in physical activity.

According to the coaches, successful recruitment and retention of coaches depends on whether the local club authorities, other FCPC coaches, and healthcare professionals receive support during the start-up phase. Many FCPC clubs form networks across clubs, which the coaches find valuable because they provide the opportunity to exchange experiences with other coaches.

Lastly, the coaches advise non-professionals who are considering coaching clinical populations. They pointed out that potential coaches primarily need to reflect on whether they are willing to invest their time and that they need to refuse to feel intimidated by the thought that they do not know exactly what to do:

C11 Gain as much knowledge as you possibly can, and then throw yourself into it because then you learn what to do.

## Discussion

This exploration of the experiences that non-professional soccer coaches have in delivering community-based soccer training for men with prostate cancer contributes with unique insights into what it means for non-health professionals to have responsibility for exercise training for a clinical population in the community (i.e., non-clinical setting).

One interesting finding was that the FCPC coaches found that the delivery of soccer training for the clinical subgroup of men with prostate cancer was easier in practice than in theory. They felt adequately prepared by the one-day FCPC course but emphasized that they did not truly discover their coaching role or establish team norms until they met the players. This finding is in line with situated learning theory. Educational theorist and practitioner Etienne Wenger [37], known for his theory on community of practice, conceptualizes learning as a social process in which learning does not take place by listening to a master, but among the participants as they regularly interact in the social context in which the intervention takes place [38]. As such, the FCPC soccer teams can be viewed as communities of practice that provide the opportunity for everyone involved to develop, for example, skills, management resources, and social capital. Some coaches stressed the importance of exchanging ideas with health professionals and peer coaches, indicating that the teams are not to be viewed as isolated communities but as part of a broader social system that involves several communities. Thus, the knowledge acquired during the one-day course, which the coaches believe should be mandatory if lay people are to coach clinical populations, is just one facet of being coach, as indicated by our findings, which show that learning also takes place when the coaches act and interact during practices. On the pitch and in the clubhouse, they learn to define their coaching role and acquire new skills of value in providing security and supporting exercise adherence in a clinical population. Of importance, the finding that the coaches identified social community as the most important aspect of participation in FCPC and therefore considered facilitating camaraderie a prominent task is in accordance with two previous studies investigating the experiences of FCPC players (i.e. men with prostate cancer) [39, 40]. These studies found, that the players conveyed camaraderie and team spirit as the most important benefit to their participation in FCPC, as well as to their continuous motivation to participate in FCPC [39, 40].

Another notable finding of this study was the relatively long-term retention of coaches in the FCPC program, as retaining volunteer coaches is often a challenge [41]. The coaches’ experiences showed that they, through their involvement in FCPC, were enriched not only by their camaraderie and shared experiences with the players, but by the broader perspective on life they gained, which is consistent with a study showing that involvement in volunteer work and nonprofit organizations may lead to an increase in quality of life [42]. Moreover, this finding suggests that the coaches experience transformative learning, i.e. learning that fundamentally changes the perspective of the individual [43], which may explain why they feel that they grow as human beings, resulting in a lasting sense of belonging and interest.

However, this study also shows that interventions such as FCPC face important threats to their sustainability. Besides having to coach the clinical population, the coaches interviewed in the current study also had to dedicate a substantial amount of time to secure funding and recruit players. A mixed-method study of why people leave volunteer and nonprofit organizations showed that, while people overall were content and pleased with their volunteer work, they often decide to quit due to work overload and burnout [44]. As a result, it seems imperative that organizations must establish effective strategies to ensure that non-professional coaches can focus their time on coaching alone, as this is what they are truly passionate about.

Of importance in this regard, most FCPC coaches had a prior or current affiliation with the club where they coached FCPC. According to Henriksen et al. [45], 79% of people who volunteer in Denmark are a member of the organization they volunteer for, ascribing institutional trust as an important factor when recruiting volunteers. Thus, the results of this study indicate that in several respects it can be advantageous to link exercise training of a clinical population in the community to an already-existing sports club to secure lay coaches to deliver the exercise intervention and to make the clinical group an integral part of the club. This also stresses the relevance of establishing and promoting a network for FCPC coaches that would allow them to share experience and knowledge across clubs. During the interviews the coaches made what appears to be a warranted appeal for better infrastructure, including more formal communication channels and referral pathways between the lay coaches and clinicians (i.e., trained health professionals in charge of and responsible for monitoring the disease and treatment). Improved infrastructure is pivotal not only in relation to the sustainability of the teams, but also with regard to protecting the players and maintaining their safety. According to Hardcastle et. al. [15], medical and surgical oncologists are in an ideal position to offer guidance on health behavior change because patients trust their authority. Based on our results, we believe that more formal interaction pathways and communication channels between coaches/clubs and clinicians/hospitals would likely also take into consideration the coaches’ awareness of the limitations of their knowledge and the boundaries of their responsibilities. This includes not using coaches to screen candidates for recruitment.

Finally, our findings raise the important issue as to whether players who have a cancer diagnosis should be mixed with healthy players and/or players with other types of chronic disease. In the current study, the inclusion of non-clinical participants was largely done to secure enough players to constitute a soccer team and thus to enhance the players’ commitment to the activity. According to the coaches of mixed teams, the players with prostate cancer show up to practice already wearing their sports clothes, most likely to hide that they use adult incontinence briefs. In teams exclusively comprising players with prostate cancer, the team shares the understanding that they can openly discuss and joke about this and other disease-related issues. More research on the (dis)advantages of diagnosis-specific community-based exercise programs appears warranted.

### Methodological Considerations

Using inductive content analysis allowed us to capture the intended objective of the study, one underexplored by previous research. Despite applying a convenience sample to secure enough participants, variability in the sample was obtained with the representation of coaches from FCPC teams in both large and small cities across Denmark. The coaches, who represented all ages, comprised both prior and current FCPC coaches, in addition to displaying a wide variation in how long they provided FCPC soccer training. A limitation is that we only interviewed male coaches as it cannot be ruled out that female coaches might have different views and experiences than those described in this study. However, we believe that these findings may be transferable to the delivery of community-based exercise interventions in other clinical populations by non-professionals with no prior experience or any training as a healthcare professional.

The open-ended questions provided rich, detailed descriptions that reflected the variation in individual experiences, while the focused questions facilitated the identification of recommendations of relevance for future planning, implementation, and dissemination of community-based training programs for clinical populations supervised by non-professionals. Due to the current COVID-19 pandemic and the large geographic distance between the FCPC clubs, interviews were conducted via telephone. The researchers found that the telephone interviews, which were of sufficient length, served the objective of the study and resulted in sufficient information power. The interview data was rich in detail, furnishing detailed information from the coaches on their personal knowledge, the skills they acquired, and what they experienced in relation to delivery of the FCPC program. Interviews were conducted and transcribed in Danish before being translated into English by a native-speaking professional translator to ensure their accuracy.

We acknowledge that the findings of this study, as with other qualitative research, are influenced by our own preconceptions. To enhance credibility, the researchers continuously reflected on and discussed their own preconceptions relating to the objective of the study throughout the entire research process to ensure that the findings are devoid of investigator interference as much possible [46].

### Implications for Practice

The findings in this study showed that lay coaches in charge of the delivery of soccer training for men with prostate cancer appreciate and value being taught about the challenges and needs of men with prostate cancer while also acknowledging and appreciating the need to develop and adjust their own coaching role during practice and while interacting with the players. Based on our findings, we propose the following recommendations for supporting the safe implementation and sustainability of community-based exercise programs that cater to clinical populations supervised by non-professional coaches:

- Ensure that a coaching course that specifically provides insight into the illness that the players are undergoing treatment for is available and mandatory
- Establish networks for the exchange of experiences between coaches across clubs, including the opportunity to provide mutual support and swap ideas (when dealing with difficult situations and events)
- Establish formal, stable communication channels between volunteer coaches and clinicians, and relieve coaches from any responsibility of recruitment’
- Ensure a financially sustainable model and relieve coaches of any responsibility for communication on financing

## Conclusions

In conclusion, the research findings in this study show that it is easier in practice than in theory for non-professional soccer coaches to deliver community-based soccer training for men with prostate cancer. Initial concerns about how to deliver soccer training to a clinical group disappeared once the coaches met the players and developed their own personal coaching style. The coaches described few downsides to coaching a clinical population and felt they were balanced out by transformative experiences due to engaging with players, ultimately leading to a broader perspective on life.

## Supplementary Information

**Additional file 1: Table S1.** COREQ checklist.

**Additional file 2: Table S2.** Interview guide

## Data Availability

Please contact the authors to request data.

## Abbreviation

FCPC: Football Club Prostate Community
