# Supplementary material for: Easier in Practice Than in Theory: Experiences of Coaches in Charge of Community-Based Soccer Training for Men with Prostate cancer—A Descriptive Qualitative Study
Source: Sports Med Open. 2022 Mar 3;8:28. doi: 10.1186/s40798-022-00424-z (PMC8892393; doi:10.1186/s40798-022-00424-z)
Supplement: Supplementary file 1 — Additional file 1: Table S1. COREQ checklist. [file 40798_2022_424_MOESM1_ESM.docx]

*Easier in Practice than in Theory – Experiences of Coaches in Charge of Community-Based Soccer Training for Men with Prostate Cancer: A Descriptive Qualitative Study*. Sports Medicine – Open. Kickan Roed^1,2*^, Eik Dybboe Bjerre^2^, and Julie Midtgaard^1,2,3^

* Correspondence: [kickan.roed@regionh.dk](mailto:kickan.roed@regionh.dk)

Mental Health Center, Glostrup, Copenhagen University Hospital – Mental Health Services CPH, Forskningsenheden, Nordstjernevej 41, DK-2600 Glostrup, Denmark

^2^ The University Centre for Health Research, Copenhagen University Hospital, Rigshospitalet, Blegdamsvej 9, DK-2100 Copenhagen Ø, Denmark

^3^ Department of Clinical Medicine, University of Copenhagen, Blegdamsvej 3B, DK-2200 Copenhagen N, Denmark

**Additional file 1**

Please note that the COREQ checklist originally operates with reporting items according to which page number they can be found on. The page numbers in this supplementary file refer to the original manuscript. An added row to the checklist provides information as to which section and heading of the article specific items are reported in.

**Table S1.** Consolidated criteria for reporting qualitative studies (COREQ): 32-item checklist

Developed from:

Tong A, Sainsbury P, Craig J. Consolidated criteria for reporting qualitative research (COREQ): a 32-item checklist for interviews and focus groups. *International Journal for Quality in Health Care*. 2007. Volume 19, Number 6: pp. 349 – 357

| **Topic/ Item No.** | **Guide Questions/Description** | **Reported on Page No.** | **Section and heading** |
| --- | --- | --- | --- |
| **Domain 1: Research team and reﬂexivity** |  |  |  |
| *Personal Characteristics* |  |  |  |
| 1. Interviewer/facilitator | Which author/s conducted the interview or focus group? | 6 | Methods, under ‘Data Collection’ |
| 2. Credentials | What were the researcher’s credentials? E.g. PhD, MD | N/A | N/A |
| 3. Occupation | What was their occupation at the time of the study? | 6, 7 | Methods, under ‘Data Collection’ |
| 4. Gender | Was the researcher male or female? | 6, 7 | Methods, under ‘Data Collection’ |
| 5. Experience and training | What experience or training did the researcher have? | 6 | Methods, under ‘Data Collection’ |
| *Relationship with participants* |  |  |  |
| 6. Relationship established | Was a relationship established prior to study commencement? | 6 | Methods, under ‘Data Collection’ |
| 7. Participant knowledge of the interviewer | What did the participants know about the researcher? e.g. personal goals, reasons for doing the research | N/A | N/A |
| 8. Interviewer characteristics | What characteristics were reported about the inter viewer/facilitator? e.g. Bias, assumptions, reasons, and interests in the research topic | 25 | Discussion, under ‘Methodological Considerations’ |
| **Domain 2: study design** |  |  |  |
| *Theoretical framework* |  |  |  |
| 9. Methodological orientation and Theory | What methodological orientation was stated to underpin the study? e.g. grounded theory, discourse analysis, ethnography, phenomenology, content analysis | 8 | Methods, under ‘Data Analysis’ |
| *Participant selection* |  |  |  |
| 10. Sampling | How were participants selected? e.g. purposive, convenience, consecutive, snowball | 5-6 | Methods, under ‘Sampling’ |
| 11. Method of approach | How were participants approached? e.g. face-to-face, telephone, mail, email | 5 | Methods, under ‘Sampling’ |
| 12. Sample size | How many participants were in the study? | 6, 7, 10 | Methods, under ‘Sampling’ and ‘Data Collection’  Results, under ‘Sample Characteristics’ |
| 13. Non-participation | How many people refused to participate or dropped out? Reasons? | 6 | Methods, under ‘Sampling’ |
| *Setting* |  |  |  |
| 14. Setting of data collection | Where was the data collected? e.g. home, clinic, workplace | 6 | Methods, under ‘Data Collection’ |
| 15. Presence of non-participants | Was anyone else present besides the participants and researchers? | 6, 7 | Methods, under ‘Data Collection’ |
| 16. Description of sample | What are the important characteristics of the sample? e.g. demographic data, date | 10, Table 2 | Results, under ‘Sample Characteristics’ |
| *Data collection* |  |  |  |
| 17. Interview guide | Were questions, prompts, guides provided by the authors? Was it pilot tested? | 7, Additional file 2: Table S2 | Methods, under ‘Data Collection’ |
| 18. Repeat interviews | Were repeat inter views carried out? If yes, how many? | N/A | N/A |
| 19. Audio/visual recording | Did the research use audio or visual recording to collect the data? | 7 | Methods, under ‘Data Collection’ |
| 20. Field notes | Were ﬁeld notes made during and/or after the interview or focus group? | N/A | N/A |
| 21. Duration | What was the duration of the interviews or focus group? | 7 | Methods, under ‘Data Collection’ |
| 22. Data saturation | Was data saturation discussed? | 6 | Methods, under ‘Sampling’ |
| 23. Transcripts returned | Were transcripts returned to participants for comment and/or correction? | N/A | N/A |
| **Domain 3: analysis and ﬁndings** |  |  |  |
| *Data analysis* |  |  |  |
| 24. Number of data coders | How many data coders coded the data? | 8 | Methods, under ‘Data Analysis’ |
| 25. Description of the coding tree | Did authors provide a description of the coding tree? | 8-9 | Methods, under ‘Data Analysis’ |
| 26. Derivation of themes | Were themes identiﬁed in advance or derived from the data? | 8 | Methods, under ‘Data Analysis’ |
| 27. Software | What software, if applicable, was used to manage the data? | 8 | Methods, under ‘Data Analysis’ |
| 28. Participant checking | Did participants provide feedback on the ﬁndings? | N/A | N/A |
| *Reporting* |  |  |  |
| 29. Quotations presented | Were participant quotations presented to illustrate the themes/ﬁndings? Was each quotation identiﬁed? e.g. participant number | 12-20 | Results, under ‘Analysis of Findings’ |
| 30. Data and ﬁndings consistent | Was there consistency between the data presented and the ﬁndings? | 12-20 | Results, under ‘Analysis of Findings’ |
| 31. Clarity of major themes | Were major themes clearly presented in the ﬁndings? | 11-20, Table 3 | Results, under ‘Analysis of Findings’ |
| 32. Clarity of minor themes | Is there a description of diverse cases or discussion of minor themes? | 12-20 | Results, under ‘Analysis of Findings’ |
