# Supplementary material for: Easier in Practice Than in Theory: Experiences of Coaches in Charge of Community-Based Soccer Training for Men with Prostate cancer—A Descriptive Qualitative Study
Source: Sports Med Open. 2022 Mar 3;8:28. doi: 10.1186/s40798-022-00424-z (PMC8892393; doi:10.1186/s40798-022-00424-z)
Supplement: Supplementary file 2 — Additional file 2: Table S2. Interview guide [file 40798_2022_424_MOESM2_ESM.docx]

*Easier in Practice than in Theory – Experiences of Coaches in Charge of Community-Based Soccer Training for Men with Prostate Cancer: A Descriptive Qualitative Study*. Sports Medicine – Open. Kickan Roed^1,2*^, Eik Dybboe Bjerre^2^, and Julie Midtgaard^1,2,3^

* Correspondence: [kickan.roed@regionh.dk](mailto:kickan.roed@regionh.dk)

Mental Health Center, Glostrup, Copenhagen University Hospital – Mental Health Services CPH, Forskningsenheden, Nordstjernevej 41, DK-2600 Glostrup, Denmark

^2^ The University Centre for Health Research, Copenhagen University Hospital, Rigshospitalet, Blegdamsvej 9, DK-2100 Copenhagen Ø, Denmark

^3^ Department of Clinical Medicine, University of Copenhagen, Blegdamsvej 3B, DK-2200 Copenhagen N, Denmark

**Additional file 2**

**Table S2.** Interview guide

| The first questions concern your role as a coach.   1. How do you perceive your role as a coach for an FC Prostate team?   Prompts:   - - - What do you consider to be your key task as a coach for the team?     - What do you emphasize during the training?     - How does this match with the coaching manual you have been taught as an FC Prostate coach - have you stuck to it, or have you found something that works better?  1. What made you want to be a coach for an FC Prostate team?   Prompt:   - - - What thoughts did you have before agreeing to become a coach at FC Prostate?   The next questions concern the fact that the players you coach in FC Prostate have been or are being treated for prostate cancer.   1. What role would you say the illness has played in relation to the soccer training, between you and the participants, and among the participants?     Prompts:   - - - How did you handle incidents and/or questions related to the players' illness, such as side effects, worsening of the illness, or death?     - How did you and the players handle it as a group?  1. Was it different for you, or were things different for you, when coaching people with an illness compared to people you have previously coached?   Prompts:   - - - Has it given rise to particular considerations that these players are ill?     - Did coaching a group of people who are ill give rise to insecurities or worries for you?     - Do you feel a distinct obligation to the men in FC Prostate compared to other people you have coached (because the players have been or are being treated for cancer)?  1. Did you experience lacking skills or knowledge that were not covered by the FC Prostate coaching program?   Prompts:   - - - Have you sought out any form of support regarding how to manage your role as an FC Prostate coach?     - Where?     - With whom?  1. In your experience, how did the soccer club support the team and your effort as a coach?   Prompt:   - - - What does this mean to you?   The following question concern injuries and prevention of injuries.   1. How do you handle player safety and prevention of sports injuries?   Prompt:   - - - Do you take any particular action to avoid sports injuries?     - Who is responsible for ensuring that the soccer training takes place in a safe manner (to avoid injuries)?   The final questions involve good experiences you have had, and which advice and recommendations you want to pass on if initiatives such as FC Prostate are to be sustainable.   1. Were there things you have been particularly happy about when coaching the men in FC Prostate?   Prompts:   - - - Why exactly has this been associated with particular joy?     - What does this mean to you?     - How would you explain that this has been possible to achieve?     - What caused it?     - Have you done/are you doing anything special to achieve it?  1. Which recommendations or good advice would you, with your experience, pass on to others who are considering becoming a coach of a team where the players have an illness?   Prompts:   - - - What is the most important experience you have gained as a coach for FC Prostate?     - What is important to be aware of if FC Prostate and similar initiatives are to be disseminated?     - What is the biggest threat to the proliferation of initiatives like FC prostate?     - Do you have views on how the recruitment/referral of new players can be secured/improved?   Do you have anything you want to add regarding your experience of coaching people with prostate cancer that I have not covered? |
| --- |
| Demographic data  - Age?  - Experience as a soccer player?  - Experience as a soccer coach before FC Prostate?  - Number of months/years as a coach in FC Prostate?  - Coach in city of >200.000 inhabitants or town of <60.000 inhabitants? |
